# Supplementary material for: A mixed-methods study of challenges and benefits of clinical academic careers for nurses, midwives and allied health professionals
Source: BMJ Open. 2019 Oct 7;9(10):e030595. doi: 10.1136/bmjopen-2019-030595 (PMC6797317; doi:10.1136/bmjopen-2019-030595)
Supplement: Supplementary data [file bmjopen-2019-030595supp001.pdf]

East Midlands Clinical-Academic Practitioner Network

Clinical Academic Survey

Clinical academic survey

The East Midlands Clinical Academic Steering Group are hoping to demonstrate the impact of clinical academic training programmes so that future cohorts are able to benefit from the same opportunities that you have had.

With this in mind, we would be extremely grateful if you could take a few minutes to complete this questionnaire in Part 1 and also, if possible, to answer the additional questions in Part 2 (approx. 10 minutes). Part 3 is for people who are currently on a clinical academic training programme.

We appreciate that some work on this has already been undertaken by others, so if you have already taken part in a similar survey, we do not expect you to repeat the information.

Please be assured that all of your responses will be treated confidentially. Also, any quotes from the data that we may use when disseminating the results, will be anonymised.

All returned responses will be entered into a **prize draw** for a chance to **win a book of your choice up to a value of £50**. Please return the form by **20th April, 2018** and provide your name and email address here so that we can contact the prize winner.

Trusson D, *et al. BMJ Open* 2019; 9:e030595. doi: 10.1136/bmjopen-2019-030595

Thank you very much in advance for your help. Good luck in the prize draw!

Part 1

Age group *Optional*

- ○ 20-30
- ○ 31-40
- ○ 41-50
- ○ 51+

How would you describe your gender?

- ○ Male
- ○ Female
- ○ Other
- ○ Prefer not to say

Current employment

- ☐ NHS
- ☐ University
- ☐ Both NHS and University
- ☐ Other employer

Please give the name(s) of your employer(s)

Trusson D, *et al*. *BMJ Open* 2019; 9:e030595. doi: 10.1136/bmjopen-2019-030595

Please give your job title(s) and professional role (e.g assistant professor and nurse)

Are you employed:

- Full-time
- Part-time

At what level? (e.g. AFC Band or HEI scale)

Trusson D, *et al*. *BMJ Open* 2019; 9:e030595. doi: 10.1136/bmjopen-2019-030595

Training programmes

Which of these clinical academic training programmes have you undertaken/are you undertaking? Please tick all that apply:

- ☐ Bronze fellowship
- ☐ Silver fellowship
- ☐ MARM
- ☐ NIHR funded MARM
- ☐ Masters in Clinical Research Studentship
- ☐ Other Masters programme
- ☐ Professional Doctorate
- ☐ NIHR funded PhD
- ☐ PhD not funded by NIHR
- ☐ Gold fellowship
- ☐ Other

Where did you undertake each training? (please list details for all courses that you have done) ✱ *Required*

How was your PhD funded?

If you selected Other, please specify: **Was it clinical academic training or another type of training?** Please provide details such as training provider; course completed/predicted completion date; qualification:

Trusson D, *et al. BMJ Open* 2019; 9:e030595. doi: 10.1136/bmjopen-2019-030595

Which is the last training that you have done and when did it/will it finish? (month/year)

What were your reasons for stopping training at that point?

For current PhD students: When did you start your PhD? (month/year)

Is it NIHR funded?

- Yes
- No

If not, how is your PhD funded?

Where are you on the PhD journey?

Trusson D, *et al. BMJ Open* 2019; 9:e030595. doi: 10.1136/bmjopen-2019-030595

- ☐ Year 1
- ☐ Year 2
- ☐ Year 3
- ☐ Year 4
- ☐ Year 5
- ☐ Year 6
- ☐ Thesis pending

What is your anticipated end date? (month/year)

Trusson D, *et al*. *BMJ Open* 2019; 9:e030595. doi: 10.1136/bmjopen-2019-030595

Please give brief details below of what you have done as a consequence of your funding/training:

Have you changed your employment in any way? For example, new roles; grades etc?

Please describe ways in which your training has had an impact or changed your clinical practice:

Have you disseminated your research:

- [ ] Locally
- [ ] Nationally
- [ ] Both

Please provide details of how you have disseminated your research (e.g. publications, conference presentations/posters). If possible, please provide links or cut and paste details.

Trusson D, *et al. BMJ Open* 2019; 9:e030595. doi: 10.1136/bmjopen-2019-030595

Have you had any successful grant applications or other collaborations?

- Yes
- No

If so, please provide details (e.g. the funding body, research project, grant reference (if any), and whether you were the lead or co-applicant)

Thank you for the answers you have provided so far. We would be grateful if you could also take a few minutes to complete some or all of the questions in part 2. Current clinical academics are also invited to complete part 3. Otherwise, please skip to the finish at the end of part 3 to submit your survey.

- Proceed to part 2
- Skip to part 3

Trusson D, *et al. BMJ Open* 2019; 9:e030595. doi: 10.1136/bmjopen-2019-030595

Part 2

In this part of the questionnaire, we want to find out what the term ‘clinical academic’ means to you, and how the clinical academic training you have received has influenced your career.

The description ‘clinical academic’ is varied. We want to try to find a way to best explain what the role is. Please explain what you understand by the title ‘clinical academic’:

Do you consider yourself to be a clinical academic?

- Yes
- No

If so why? (for example, do you have funded research and clinical sessions?)

How has the clinical academic training you have received helped you in your career and/or clinical practice?

Trusson D, *et al. BMJ Open* 2019; 9:e030595. doi: 10.1136/bmjopen-2019-030595

How did you overcome any challenges in achieving success and/or progressing to the next level in your clinical academic career?

What types of support did you find most helpful during your clinical academic training?

Have your goals changed during your training?

- Yes
- No

If so, please explain how and why your goals have changed:

Trusson D, *et al. BMJ Open* 2019; 9:e030595. doi: 10.1136/bmjopen-2019-030595

Are there any resources that you found beneficial in developing your clinical academic role which you would be happy to share for the benefit of others?

These might include, for example, a job description or a report which was helpful in gaining the support of your managers for your training.

If you have any resources that you think might be useful, please send them as an attachment to the following email: diane.trusson@nottingham.ac.uk . Alternatively, please give link(s) to electronic resources here:

What advice would you give to people who are considering undertaking training and embarking on a clinical academic career?

Is there anything else that you want to add?

If you are currently on a clinical academic training programme (e.g. MRes/MARM/Bronze or Silver scholar/PhD/Post-doc), please complete the questions in Part 3. Otherwise, please press 'next' to finish on the final page.

Trusson D, *et al. BMJ Open* 2019; 9:e030595. doi: 10.1136/bmjopen-2019-030595

Part 3. If you are currently on a clinical academic training programme e.g. MRes/MARM/Bronze or Silver scholar/PhD/Post-doc

Would you be interested in taking part in a focus group to discuss your experiences?

- Yes
- No

If so, please provide your contact details here:

We would also like to develop some case studies to demonstrate the impact that clinical academic training can have both in relation to your career and your clinical practice. Would you be willing to be interviewed about your experiences?

- Yes
- No

If so, please provide your contact details here:

Trusson D, *et al. BMJ Open* 2019; 9:e030595. doi: 10.1136/bmjopen-2019-030595

Thank you for taking the time to complete this survey, your responses are very important to us.

Trusson D, *et al*. *BMJ Open* 2019; 9:e030595. doi: 10.1136/bmjopen-2019-030595
