# Supplementary material for: A mixed-methods study of challenges and benefits of clinical academic careers for nurses, midwives and allied health professionals
Source: BMJ Open. 2019 Oct 7;9(10):e030595. doi: 10.1136/bmjopen-2019-030595 (PMC6797317; doi:10.1136/bmjopen-2019-030595)
Supplement: Supplementary data [file bmjopen-2019-030595supp002.pdf]

1. Can you tell me a bit about your experience of being a clinical academic?
2. What was/is your motivation for doing clinical academic training?
3. In what ways do you think your training has helped you to develop as a clinician?
4. What are you doing differently?
5. How have you brought your research into your clinical practice – what have you done because of your training?
6. Can you give me examples of ways that your clinical practice has changed as a result of your training?
7. How have your changes been beneficial? Do you have any evidence of benefits to practice?
8. Research project impact
9. Influence on the practice of others?
10. Is there anything else you want to add?
